# Supplementary material for: Time-series transcriptome analysis identified differentially expressed genes in broiler chicken infected with mixed Eimeria species
Source: Front Genet. 2022 Aug 8;13:886781. doi: 10.3389/fgene.2022.886781 (PMC9393255; doi:10.3389/fgene.2022.886781)
Supplement: Supplementary file 2 [file DataSheet1.ZIP › 7dpi_KEGG.Gsea.1625115961950/KEGG_COLORECTAL_CANCER.html]

Details for gene set KEGG\_COLORECTAL\_CANCER[GSEA]

|  || Dataset | TMM\_7dpi\_gct\_format\_7dpi\_gct\_format.Class\_4dpi.cls #PC\_versus\_NC.Class\_4dpi.cls #PC\_versus\_NC\_repos |
| Phenotype | Class\_4dpi.cls#PC\_versus\_NC\_repos |
| Upregulated in class | 0 |
| GeneSet | KEGG\_COLORECTAL\_CANCER |
| Enrichment Score (ES) | -0.47156206 |
| Normalized Enrichment Score (NES) | -1.565583 |
| Nominal p-value | 0.008080808 |
| FDR q-value | 0.09738446 |
| FWER p-Value | 0.8206 |
Table: GSEA Results Summary

  

Fig 1: Enrichment plot: KEGG\_COLORECTAL\_CANCER      
 Profile of the Running ES Score & Positions of GeneSet Members on the Rank Ordered List

  

| SYMBOL | TITLE | RANK IN GENE LIST | RANK METRIC SCORE | RUNNING ES | CORE ENRICHMENT || 1 | CYCS | na | 475 | 0.763 | -0.0120 | No |
| 2 | TCF7L1 | na | 1896 | 0.288 | -0.1202 | No |
| 3 | TGFB2 | na | 1954 | 0.278 | -0.1149 | No |
| 4 | CASP9 | na | 2022 | 0.268 | -0.1107 | No |
| 5 | BCL2 | na | 2059 | 0.261 | -0.1042 | No |
| 6 | BIRC5 | na | 2944 | 0.133 | -0.1733 | No |
| 7 | RAC1 | na | 2998 | 0.126 | -0.1731 | No |
| 8 | RHOA | na | 3449 | 0.068 | -0.2083 | No |
| 9 | JUN | na | 4404 | -0.040 | -0.2866 | No |
| 10 | RAF1 | na | 5142 | -0.116 | -0.3440 | No |
| 11 | MAP2K1 | na | 5188 | -0.120 | -0.3434 | No |
| 12 | AXIN2 | na | 5268 | -0.128 | -0.3454 | No |
| 13 | TGFB1 | na | 5297 | -0.130 | -0.3430 | No |
| 14 | FOS | na | 5340 | -0.134 | -0.3416 | No |
| 15 | RAC3 | na | 6076 | -0.210 | -0.3954 | No |
| 16 | TGFBR2 | na | 6133 | -0.218 | -0.3922 | No |
| 17 | AXIN1 | na | 6400 | -0.243 | -0.4055 | No |
| 18 | CASP3 | na | 6448 | -0.248 | -0.4004 | No |
| 19 | MAPK8 | na | 6464 | -0.250 | -0.3926 | No |
| 20 | MLH1 | na | 6647 | -0.270 | -0.3980 | No |
| 21 | MYC | na | 6951 | -0.302 | -0.4124 | No |
| 22 | TCF7L2 | na | 7019 | -0.311 | -0.4067 | No |
| 23 | AKT3 | na | 7033 | -0.312 | -0.3964 | No |
| 24 | KRAS | na | 7206 | -0.330 | -0.3988 | No |
| 25 | MSH2 | na | 7352 | -0.347 | -0.3983 | No |
| 26 | TCF7 | na | 7360 | -0.347 | -0.3862 | No |
| 27 | TGFBR1 | na | 7506 | -0.362 | -0.3852 | No |
| 28 | MAPK9 | na | 7648 | -0.377 | -0.3833 | No |
| 29 | RAC2 | na | 7904 | -0.409 | -0.3897 | No |
| 30 | AKT1 | na | 8399 | -0.472 | -0.4138 | No |
| 31 | PIK3CG | na | 8478 | -0.481 | -0.4029 | No |
| 32 | CTNNB1 | na | 9201 | -0.583 | -0.4420 | No |
| 33 | TGFB3 | na | 9555 | -0.639 | -0.4483 | Yes |
| 34 | PIK3R2 | na | 9597 | -0.645 | -0.4283 | Yes |
| 35 | MSH6 | na | 9908 | -0.694 | -0.4290 | Yes |
| 36 | APC2 | na | 9933 | -0.699 | -0.4056 | Yes |
| 37 | SMAD3 | na | 10010 | -0.714 | -0.3859 | Yes |
| 38 | MAPK3 | na | 10320 | -0.774 | -0.3836 | Yes |
| 39 | PIK3CB | na | 10335 | -0.777 | -0.3565 | Yes |
| 40 | CCND1 | na | 10432 | -0.796 | -0.3356 | Yes |
| 41 | APC | na | 10633 | -0.841 | -0.3217 | Yes |
| 42 | PIK3CD | na | 10707 | -0.855 | -0.2967 | Yes |
| 43 | PIK3CA | na | 10920 | -0.912 | -0.2813 | Yes |
| 44 | PIK3R5 | na | 10941 | -0.918 | -0.2496 | Yes |
| 45 | PIK3R3 | na | 11045 | -0.949 | -0.2237 | Yes |
| 46 | PIK3R1 | na | 11265 | -1.023 | -0.2048 | Yes |
| 47 | MSH3 | na | 11318 | -1.044 | -0.1711 | Yes |
| 48 | LEF1 | na | 11388 | -1.076 | -0.1377 | Yes |
| 49 | APPL1 | na | 11675 | -1.222 | -0.1172 | Yes |
| 50 | BRAF | na | 11724 | -1.264 | -0.0753 | Yes |
| 51 | MAPK10 | na | 12008 | -2.724 | 0.0002 | Yes |
Table: GSEA details [plain text format]

  

Fig 2: KEGG\_COLORECTAL\_CANCER      
 Blue-Pink O' Gram in the Space of the Analyzed GeneSet

  

Fig 3: KEGG\_COLORECTAL\_CANCER: Random ES distribution      
 Gene set null distribution of ES for **KEGG\_COLORECTAL\_CANCER**
